# Automated Sagittal Craniosynostosis Classification from CT Images Using Transfer Learning

**Published:** 2020-02-27

**Authors:** Lei You, Guangming Zhang, Weiling Zhao, Matthew Greives R, Lisa David, Xiaobo Zhou

**Affiliations:** 1 School of Biomedical Informatics, The University of Texas Health Science Center at Houston, Texas, USA; 2 McGovern Medical School at The University of Texas Health Science Center at Houston, USA; 3 Children’s Memorial Hermann Hospital, USA; 4 Department of Plastic and Reconstructive Surgery, Wake Forest Medical School of Medicine, Medical Center Boulevard, USA; 5 School of Dentistry, The University of Texas Health Science Center at Houston, Texas, USA; 6 Department of Integrative Biology and Pharmacology, McGovern Medical School, The University of Texas Health Science Center at Houston, Texas, USA

**Keywords:** Sagittal craniosynostosis, Transfer learning, Convolutional neural networks, Medical image analysis

## Abstract

**Purpose:**

Sagittal Craniosynostosis (CSO) occurs when the sagittal suture of a growing child’s skull is fused. Surgery is the primary treatment for CSO. Surgical treatment involves removing the affected bones and increasing the volume of the cranium by repositioning the bone segments or using external forces to guide growth. These external forces are often achieved by internal springs or external helmet therapy and depend on surgical judgment based on patient age, severity, and subtypes of CSO. Physicians usually classify CSO subtypes by examining CT images. In our previous work, we built an objective computerized system to mimic the physician’s diagnostic process based on more than 100 hand-crafted features. However, hand-crafted features-based methods have limitations in representing all aspect features of the CSO images. To improve feature extraction efficiency, classification accuracy, and reduce subjectivity in the choice of surgical techniques, in this study, we developed a deep learning-based method to learn advanced features for the classification of CSO subtypes.

**Methods:**

First, a Hounsfield Unit (HU) threshold-based method was used to segment 3D skulls from CT slices. Second, the 3D skulls were mapped to a two-dimension space by hemispherical projection to obtain binary images with a resolution of 512 × 512. These binary images were augmented to generate a new dataset for training deep convolutional neural networks. Finally, the pre-trained deep learning model was fine-tuned on the generated dataset using transfer learning method. Both training accuracy and cross-entropy curves were used to assess the performance of the proposed method.

**Results:**

Three deep convolutional neural networks were built based on the manual classification results of CSO patients by three surgeons. The classification difference between surgeons was 54%. The prediction accuracy of the three deep learning models based on the generated dataset was greater than 90%, which was higher than the accuracy from the previous models (72%). The model based on the classification results of the senior surgeon achieved the highest performance accuracy (75%) in unseen real data, compared to 25% and 37.5% for two junior surgeons, respectively.

**Conclusion:**

Our experimental results show that deep learning is superior to the hand-crafted feature-based method for sagittal CSO classification. The performance of deep learning models still depends on the quality of the original data. The classification variability of physicians can result in differential model outputs. When given more sagittal CSO images with proper annotations, the deep learning-based models can be more stable, approximate the diagnosis performance of physicians and have the potential to reduce the inter-observer variability thereby providing clinical insight into research and the treatment selection in patients with CSO.

## Introduction

Craniosynostosis (CSO) is an extremely serious birth defect that involves premature fusion [1–3] of one or more sutures on a baby’s skull, affecting 1 in 2,000 new births. CSO can be classified into many subtypes based on suture fusion, such as sagittal, coronal, metopic, lambdoid, and other possible combinations of fused sutures. Sagittal CSO is the most common sub-type of CSO, accounting for 40% to 60% of all types [4,5]. The skull of infants with CSO cannot expand uniformly, resulting in abnormal head shape. If the deformation does not provide enough space for the growing brain, intracranial hypertension may result. This condition can cause serious neurological complications such as headache, irritability, and mental developmental delay and a lower IQ. Thus, this disease should be detected early in life, both due to its cosmetic manifestations and functional consequences. Early diagnosis is crucial for management, prevention of complications, and early surgical correction. The surgery may upgrade from removing parts of the affected bones from the skull to breaking the skull into small pieces if the CSO patient takes the surgery after one-year-old.

There are several clinical ways to classify sagittal CSO [6–8]. The diagnosis of craniosynostosis mainly relies on physical examination, plain radiography, and computed tomography [2]. Physical examinations include evaluations of the calvarial shape (vertex view) and measurement of the head circumference. Plain radiography and computed tomography are used to diagnose the craniosynostosis. Kabbani et al. [2] defined sagittal synostosis by a ridged and fused sagittal suture, bitemporal narrowing and frontal, occipital bossing. Massimi et al. [7] further classified sagittal CSO into three main variants, including anterior (evident frontal bossing), posterior (occipital bones), and complete (combination). They suggested that clinical findings [8] of sagittal CSO are unequivocal and characteristical and CT scans are not necessary for all patients. If the physician believes that the patient is in severe condition, 3D CT scan should be used to reconstruct the skull surface for surgical planning and postoperative outcome evaluation. Lisa David et al. [6] provided a sagittal CSO classification system based on 3D CT slices, in which sagittal CSO was classified into four subtypes, including anterior, central, posterior and complex. The authors gave a brief description of each sub- type based on the four views of the 3D reconstructed skull.

Current treatment strategies seek to give space for brain growth and correct the head shape by adjusting the skull, including Spring- Assisted Surgery (SAS) [9–11] Pi-Plasty [12,13] and cranial vault reconstruction [10]. SAS is recognized as a safe, effective, and less invasive treatment method [13–18]. SAS can usually be completed in less than one hour and significantly reduce blood loss compared to other methods. In addition, unlike other methods, SAS can be performed in infants <6 months [19]. Patient-specific spring selection is a crucial step for CSO treatment with SAS and largely dependent on the experience of a surgeon. Important factors in the selection of the spring force include patient age, bone thickness, and subtypes of CSO. For example, sagittal CSO with an elongated occiput needs a stronger posterior spring, while an infant with no predominant characteristics typically needs a mid-range anterior and posterior spring. Specifically, different categories of sagittal CSO are treated by SAS with particular schemes, including forces and number of springs. For example, the central type of sagittal CSO with a heaped-up sagittal ridge should be treated with two springs of approximately equal strength.

Although suture fusion is a clear indication of craniosynostosis (except for metopic phenotypes), assessing the need for surgical correction relies heavily on subjective assessment of shape abnormality. The surgical procedure, whether responding to aesthetical or functional needs, attempts to obtain an adequate cranial morphology by correcting both the deformation of the cranial bones due to compensatory over-growth and the possible ridging (i.e. increased curvature) of ossified sutural regions. An objective classification system based on image processing technologies is necessary for the diagnosing the sub-types of sagittal CSO. It usually consists of a feature extraction step and a classification step. The cranial deformations on the image are transformed into image features that are independent of the physicians’ subjective diagnosis. Such a classification step usually only needs the diagnosis results of physicians and doesn’t involve the physicians’ diagnosing process. In that case, it can support useful information without the influence of personal subjective experiences.

Based on the dominant features on CT scans, surgeons can stratify sagittal CSO into four sub-types: Anterior, central, posterior, and complex. The traditionally described characteristics, such as frontal bossing and occipital lengthening, however, are inconsistently present. The fusion of individual sutures causes the abnormal deformation of infant cranial vault. Thus, how to design an objective classification system is very important. Previous studies have developed automated approaches for coarse classification of CSO [5,20–25]. However, these methods primarily considered the shapes of cranial vaults and paid little attention to the sutures themselves. Thus, these methods were failed when applied to further sub-typing sagittal CSO patients.

Some researchers tried to segment each bone and suture on the 3D surface of the skull and used the relationships among them to classify CSO. For example, many doctors and researchers have tried to find some discriminative characteristics from CT images, especially from the 3D structure of skulls [25,26] to determine CSO subtypes. Mendoza et al. [25] used statistical shape modeling to describe five cranial bones and six sutures above two cranial anatomy planes based on hand-crafted features. Ghadimi et al. [26] proposed a coupled level sets-based method for newborn skull segmentation and fontanels-sutures reconstruction. These methods have achieved good performance in CSO analysis. However, similar to the ordinary image processing, the hand-crafted features-based model has many limitations in processing high-dimension image data.

In the past few years, deep learning-based models have shown their advantages in learning the distribution of large datasets and are widely used in computer vision, speech recognition, machine translation, natural language processing and other fields [27]. Convolutional Neural Network (CNN) is one of the most widely used architectures in the field of computer vision and its variant architectures are also widely used in medical image analysis. For example, Roth et al. [28] proposed a CNN based automated pancreas location and segmentation architecture using a two steps strategy. Coudray et al. trained CNN models to classify cancer cells (adenocarcinoma, squamous cell carcinoma) and normal cells in lung pathology images. Recently, CNN based cycle generative adversarial networks were applied to segment the spleen from multi-modality images. 3D deep CNN architectures are used for brain tissues segmentation.

From the related work of craniosynostosis classification, we can see that most of the existing methods are hand-crafted feature-based. To improve feature extraction efficiency, classification accuracy, and reduce subjectivity in the choice of surgical techniques, in this study, a deep learning-based method was utilized to classify four sub-types of sagittal CSO. Considering the limited amount of data, transfer learning was adopted instead of training a new network from scratch. Although transfer learning can partly alleviate the problem of data shortage, data augmentation is still necessary. Instead of learning 3D representations of skulls, we learned representations in a 2D plane to show the effectiveness of our proposed method. Our previous 3D skull projection method [29] could supply such 2D images on which data augmentation was applied. Our experiments showed that by combining our proposed 3D image projection, data augmentation and transfer learning, our deep learning-based model achieved a test accuracy more than 90% on the generated data.

## Methods

**Figure 1:**
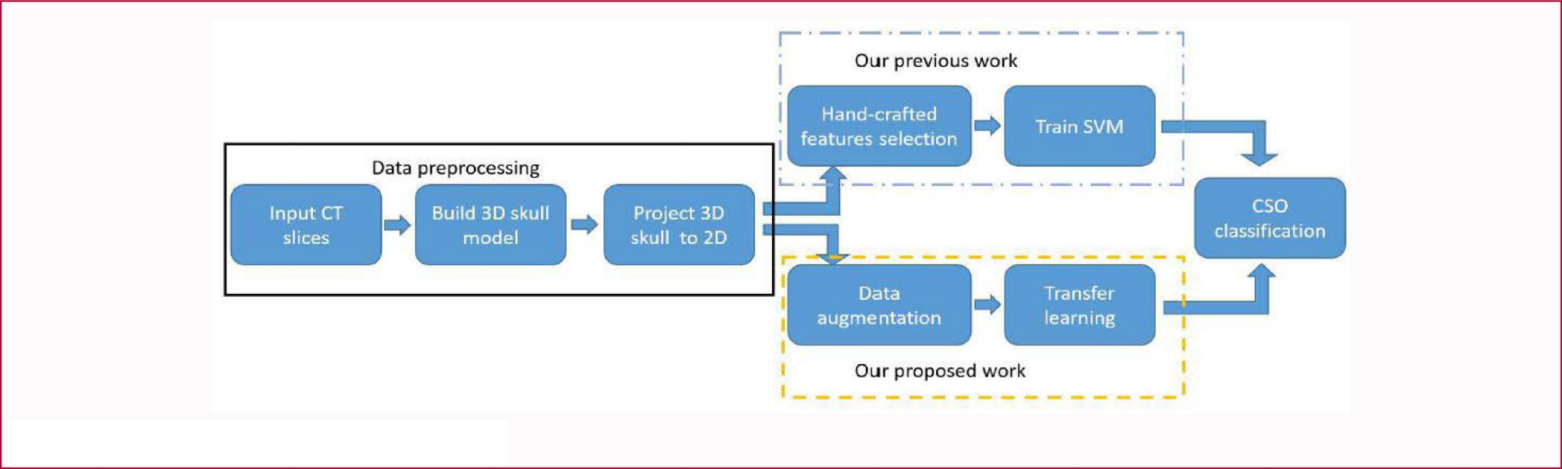
The architecture of our proposed work.

As shown in Figure 1, our proposed Sagittal Craniosynostosis classification system consists of data preprocessing and classifier training. The upper panel shows our previous classification system [29], which is based on the selected hand-craft features, extracted on the projection images of the 3D skull and a SVM classifier. In the lower panel, we applied a deep learning-based method to learn high-level representations of these projection images. In the following sections, we will describe our data augmentation and transfer learning in details.

The source codes are available upon request. Eventually it will be opened for academic use after extensively testing by different users.

### Patient cohort and dataset

**Figure 2:**
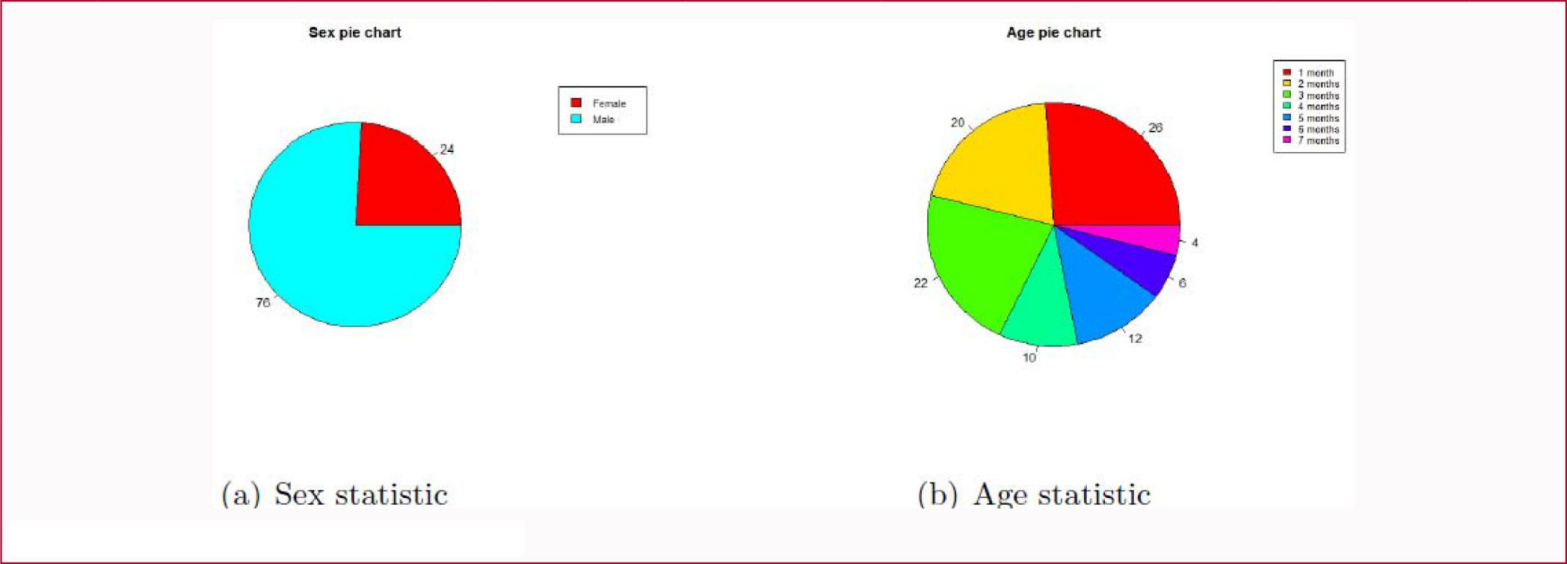
The patient demographics of our study.

A total of 50 Sagittal CSO CT cases were used in our study. Patient age and gender statistics are summarized in Figure 2. The pie charts show that most patients are boys up to four months old. Three physicians from the Department of Plastic and Reconstructive Surgery were invited to label these cases. Based on the 3D skull model generated by a commercial software from CT slices, we provided these physicians with 4 views of the 3D skull image, similar to those in Ref [6], and four options (Anterior, Central, Posterior and Complex) for annotations.

The annotated results from three physicians were collected as our automatic sagittal CSO classification system’s ground truth. There were disagreements among these physicians. One solution is based on physicians’ voting which picks up the cases having the same annotation given by at least two physicians. This solution will give us a less number of training samples which will significantly affect the training accuracy of our model. The other solution is machine learning data driven strategy in which we keep each physician’s annotation as an individual ground truth. As a result, we will have 3 groundtruth for our dataset by the second solution. Since our deep learning model needs more data to learn the distributions of sagittal CSO data, we choose the second solution to keep more paired data.

We applied both unsupervised clustering approach and supervised deep learning models (referring to the Result section) to the augmented data and raw data, to find out which physician’s annotation is closer to the real distribution of the data. As a result, we could find out whether the senior physicians diagnosis of sagittal CSO is more accurate than juniors. If that hypothesis is true, our automatic classification system built on senior physicians experience can be used both in training the junior physicians and in diagnosing the disease.

### Image pre-processing

In this section, we will show the image pre-processing progress of our system which translates the original CT slices into the binary images that can be predicted by the CNN models.

In our previous 3D skull project method (Figure 2), we projected the CT images of patient’s cranial regions (a) into a 2D binary image (c). In Figure 2b, since we selected a part of the CT image to construct the 3D skull model, only the segmented skull model is presented, which speeds up the projection process. The white areas in Figure 2c indicate the sutures or holes on the cranial bones.

**Figure 3:**
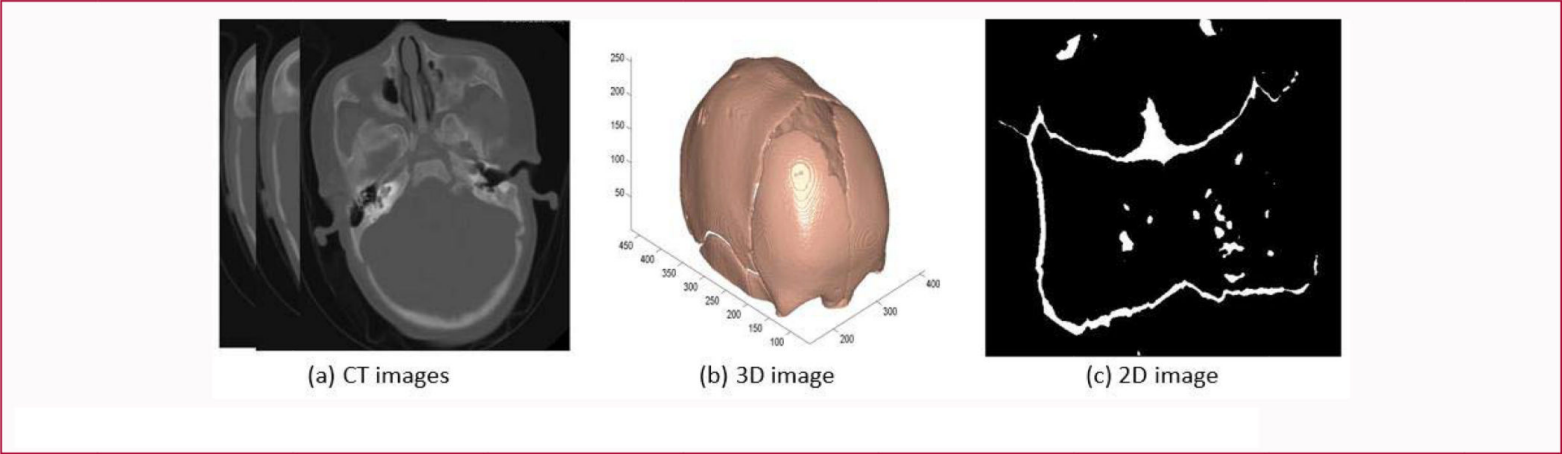
3D skull image projection processing, including original CT slices (a), 3D skull image (b) and 2D projected image (c).

In the CT images, soft tissues and bones can be segmented by a Hounsfield Units (HU) threshold because they have obviously different intensity values. For example, the soft tissues of the brain usually have a HU value less than 60, while the average HU value in bone areas is around 1000. In our study, pixels with values greater than 300 HU are recognized as bone ones of the skull. As a result, we obtained a 3D skull with gaps or holes from the CT slices. The gaps on the surface of the skull are presented as sutures (Figure 3b).

To describe the surface situations of infant skulls in a 2D plane, we used a Spherical Polar Coordinates (SPC) system to replace the original Cartesian coordinate system. We choose the center point of the bottom slice as the origin of the SPC. Under the SPC system, a skull voxel *P*_*i*_ can be specified by a triplet p(r_i_,θ_i_, φ_i_), where *r*_*i*_ is the radial distance, θ_i_ is the polar angle and φ_i_ is the azimuthal angle under the SPC coordinate system, and is projected to pixel p(m,n) according to the following formula.

$$\{\begin{array}{l} m=\left[\frac{\theta_{i}\cos\left(\varphi_{i}\right)}{\pi }N\right]+\frac{N}{2}\\ n=\left[\frac{\theta_{i}\sin\left(\varphi_{i}\right)}{\pi }N\right]+\frac{N}{2} \end{array}$$

where N is the number of rows or columns of the 2D matrix and θ_i_, $\varphi_{\text{i}}\in \left[\frac{-\pi }{2},\frac{\pi }{2}\right]$. More details can be found at Ref [29]. If the voxel belonged to the cranial bones, its projected value is set to 1, otherwise 0. After that, we can get the 2D images of skull surface like Figure 3C.

### Network training

#### Data augmentation

Data augmentation is widely used to generate more images for training the CNN based neural network in solving some medical image processing problems, such as breast cancer recognition [30] and skin cancer detection [31]. For example, in Wei et al. [30] study, the dataset was augmented 14 times through randomly rotating, scaling, mirroring samples. The reason for data augmentation is that CNNs are supposed to be invariant to the viewpoint, translations, size or even occlusions of objects in the image. The data or images before augmentation can be seen as a special view with a special size of objects in the image. Operations such as flipping rotations and scaling are used to increase the amount of our CSO projected images. We aimed to generate images sharing a similar distribution with the original suture images.

Our dataset before data augmentation consists of CT images from 50 patients. These 50 cases were classified into four subtypes by three physicians as anterior, central, posterior and complex. For example, one physician classified 14 cases as anterior sagittal CSO, 14 cases as central sagittal CSO, 9 cases as posterior sagittal CSO, 10 cases as complex sagittal CSO, and 3 cases were unlabeled. The overlap rate of the classifications from these three physicians was only 46%, which means that only 23 cases had identical labels in our dataset. There are two main problems for applying a convolutional neural network to classify the subtypes of sagittal CSO. One is that fewer images are available for training a deep model like Google Inception model. The second one is that the groundtruth (annotations) of the rest 27 cases is inconsistent. We used data augmentation and transfer learning to improve data shortage situation. For the second issue, we built three convolution neural networks for each of the groundtruth and compared their performance, including training, testing and validating accuracy.

For data augmentation, 8 cases, two cases for each subtype, were used for testing the generalizations of these three deep learning models. In the rest 42 cases, cases without annotation were removed since the physicians are not sure about which subtype some cases belong to. The remaining cases with corresponding labels were used for data augmentation and network training.

**Figure 4:**
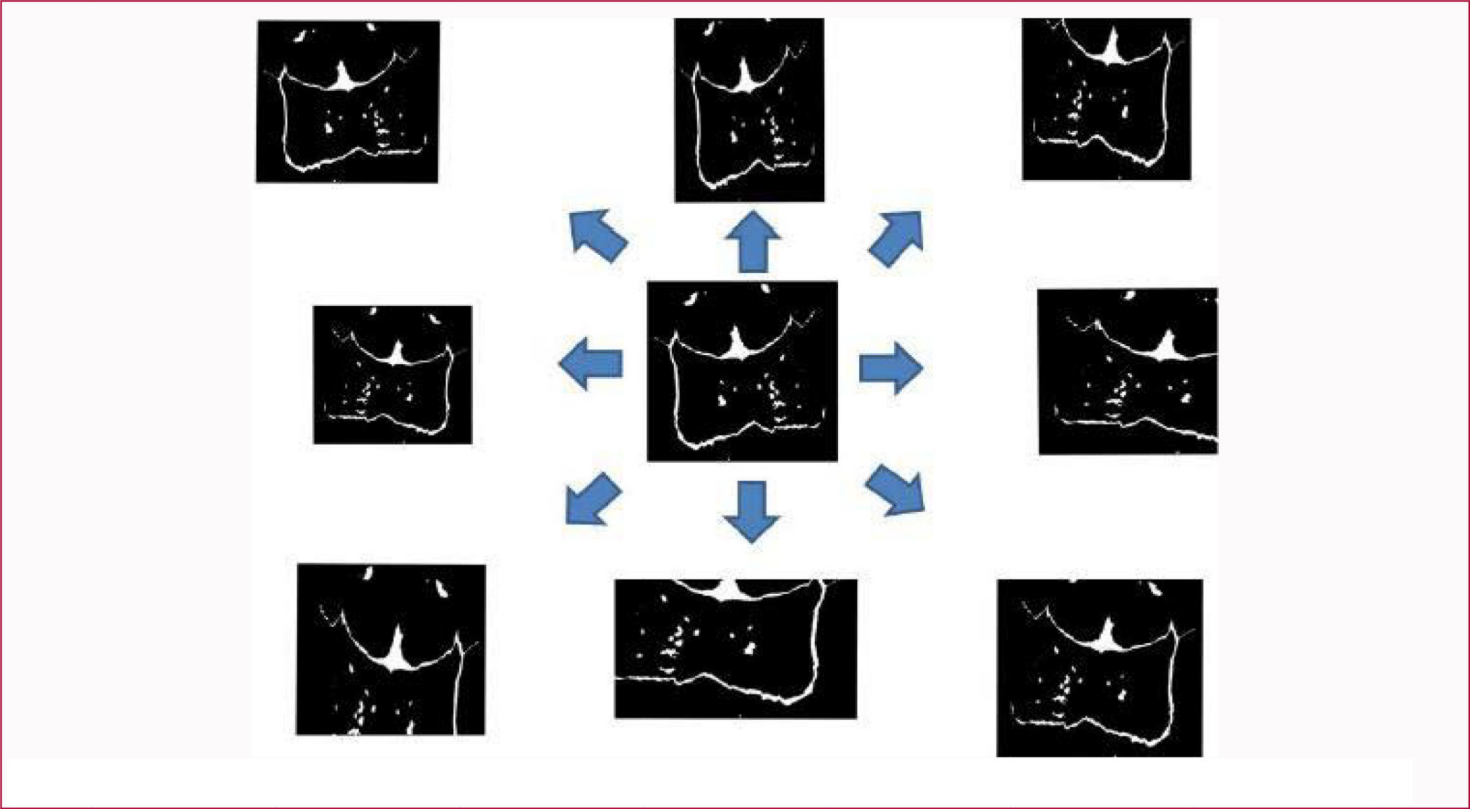
Some examples of our generated suture images. The image in the center is the original image and the images around it are the generated ones.

By adjusting data augmentation operations’ parameters, different augmentation results were achieved. For example, it shows one original projected case and its 8 surrounding augmented images in Figure 4. We hope the generated images both have different and share a similar distribution with the original one. In these eight images, the left one is a mirrored version of the original image and the upper one is re-scaled and padded by 0. The top left image is obtained by both flipping and rescaling operations. If we choose some larger factors for these operations, the generated images will look much more different and affect the fine-tuning results of the network. Thus, the transformation scale should be within a limited range. Otherwise, a generated anterior suture may be more close to a posterior one. As a result, the deep neural network may learn a biased distribution of the augmented dataset. Totally, we generated 61 images for each case in our original dataset. There were 493 anterior cases, 555 central cases, 618 posterior cases and 739 complex cases in a generated dataset according to physicians’ annotation. This dataset was then randomly divided into training, testing and validation at a ratio of 3:1:1.

#### Transfer learning

From the perspective of the development process of deep learning method, a deep and powerful neural network needs a large training dataset and lots of computation resources, such as Graphics Processing Units (GPUs), in order to succeed in a specific task. It will be very expensive to meet these requirements in the field of medical image analysis. Fortunately, Google supplies a series of transfer learning model in the model zoo under its TensorFlow architecture [32]. These models are pre-trained on large image datasets and have a good classification performance in the image processing tasks, such as object detection and object classification. Another scientific evidence of transfer learning is that deep neural networks, such as Google inception models, have learned a good representation of the basis of images. These models can quickly get convergence in a new image dataset, especially on small medical image dataset, with just fine-tuning the last several layers.

**Figure 5:**
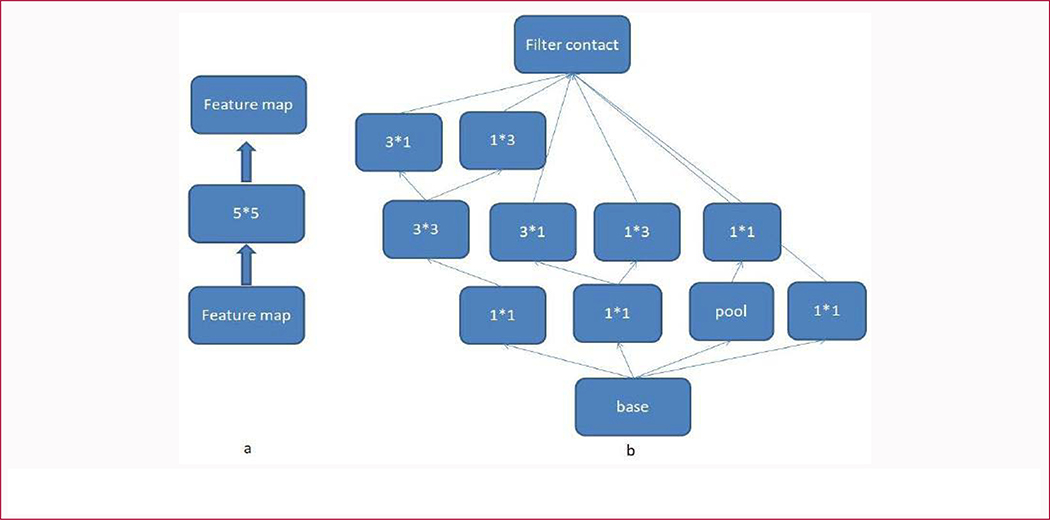
The inception module used in google inception-v3 model. In Fig a, the feature map is convolved by a 5*5 filter. In Fig b, the 5*5 filter is replaced by an inception module which not only captures the spatial information of 5*5, but also catches more fine spatial information of the previous feature map.

In our experiment, we utilized the Google Inception V3 model [33] as our transfer learning model. This model was pre-trained on the ImageNet [34] dataset, one of the biggest image datasets with labels. Compared with traditional CNNs, Google networks contain several blocks, which are named inceptions, in the architecture. The 5 × 5 convolution filter in Figure 5a was replaced by inceptions shown in Figure 5b. Large-scale of convolution filter can supply more spatial information of the feature maps, but lead to more computation cost. In the inception module, a 5 × 5 convolution filter was replaced by two connected 3 × 3 convolution filters. Furthermore, the parameters were reduced again by replacing the 3 × 3 convolution filter with 1 × 3 and 3 × 1 filters. The other inceptions in Figure 5b can supply more fine information of the feature maps.

We fine-tuned the last two layers’ parameters of the 3 model on both the originally projected images and the augmented images, keeping the other parameters fixed. The system randomly divided the images into the training, validation and testing sets. Before data augmentation, relative fewer cases in a certain class of the sagittal CSO may lead to a biased distribution of model learning data. We solved this problem by running the fine-tuning process 30 times and obtained an averaged testing accuracy over these processes. For the augmented images, we fine-tuned three models based on three physicians’ classification for 7000 iterations with a learning rate of 0.001. In the experiments section, we compared the learning results of these models.

#### Data analysis

We used TSNE method [35] to display the distributions of the generated data. As mentioned earlier, the data was generated based on each physician’s annotation. Ideally, these four subtypes should present four distinct cluster centers. If this is not the case, the less overlap between these generated data, the better the generated data. More details are shown in the result section.

We used training accuracy (each step), validation accuracy (every 10 steps) and cross- entropy to monitor the training process. After training, a final test (20% of the augmented data) was carried out to assess network performance and annotation quality. For each testing image, the network provided the probability that the image belongs to a specific subtype and compared the predicted result with its groundtruth. If the prediction matched with its groundtruth, it would be recorded as an accurate prediction. Otherwise, the cases will be recorded as a wrong prediction. The test accuracy is equal to the ratio of the number of accurate predictions to the total number of test images. At last, we compared the test accuracy of three CNN models. In addition, we also tested retained 8 real cases to measure the performance of individual CNN models.

## Experiments

We designed contrast experiments to show the effectiveness of our proposed method. First, we compared classification results before and after data augmentation. After that, we compared the hand-crafted features-based method with our deep learning-based method (after data augmentation). Finally, we compare these CNN networks on the unseen data to check their generalization or robustness.

### Data set

This study was approved by the Institutional Review Board of Wake Forest School of Medicine. The original dataset includes 50 sagittal CSO cases. Each case contains about 200 CT slices with a resolution of 512 × 512. The thickness of these slices is 0.625 mm or 1.25 mm. Within each slice, the pixel ranges from 0.43 mm to 0.41 mm with a 16-bit gray level in HU.

Three physicians (Drs. David, Branch and Sanger) manually labeled the subtypes of sagittal CSO based on the CT image data from 50 patients. We randomly picked up 8 cases (2 cases from each sub-type) for blind test and the others were used for data augmentation and network training. After data augmentation, we obtained more than 2000 images of projected sutures. The ratio of the number of images used for training, testing and validation is 3:1:1.

### Experiments design

We use Tensorflow [32] architecture to train the Google Inception-V3 model. The environment was deployed on a GPU server with two Tesla V100 gpu on it. The learning rate was set to 0.001 at beginning and Gradient Descent Optimizer was employed to train each model with 7000 steps. Training accuracy, validation accuracy, and cross entropy were used to determine the model convergence. Final test accuracy and blind test accuracy were used to test the models’ classification results from generated and real data.

We used the same initial parameters obtained from the pre-trained Google-Inception V3 model for David-CNN, Branch-CNN and Sanger-CNN. Data augmentation was conducted on all 50 projected images. According to the physicians annotation, the 50 projected images were divided into four subtypes. In each subtype, two patients were randomly selected for testing on raw images and the remaining cases were used for constructing CNN models. The ratio of training, validation and testing data is 3:1:1.

## Results

### Data augmentation results

**Figure 6:**
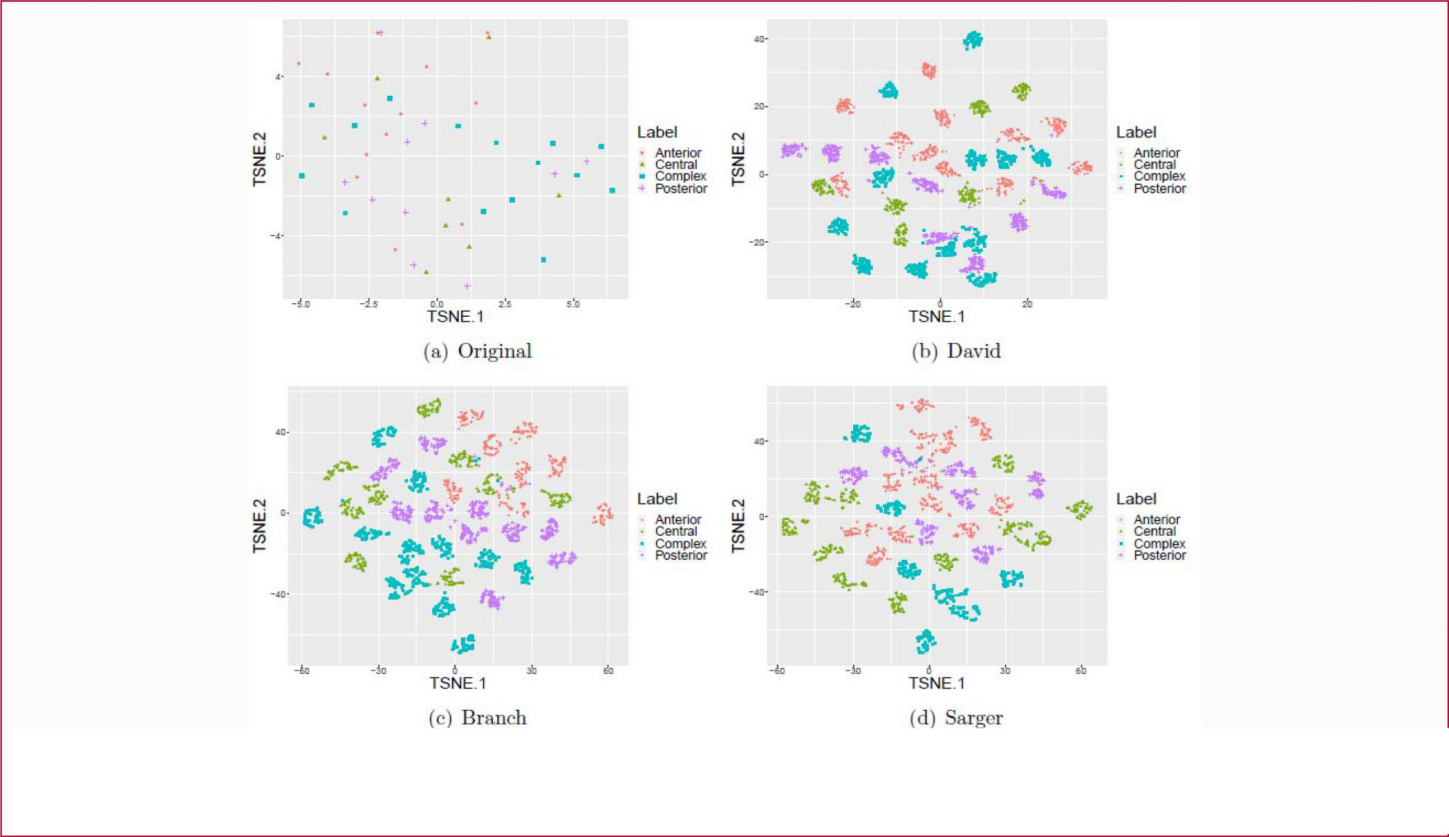
Visualization of subtype distribution of sagittal CSO before and after augmentation. Panel a shows the subtype distribution before augmentation. Panes b, c, and d show the subtype distribution after augmentation based on labels from physicians Dr. David, Branch, and Sanger, respectively. Orange, army-green, blue and purple symbols represent different subtypes of sagittal CSO.

TSNE [35] is a visualization tool for checking distributions of high dimensional data. We use this tool to visualize high-level feature vectors extracted from input images. The feature vectors were from the last fully connected layer of the network. Suture image augmentation results are shown in Figure 6. Each color/shape represents one subtype of sagittal CSO. The distribution of before augmentation is shown in Figure 6a. We can see that there is no clear clustering center for each sub-type of sagittal CSO. At the same time, there are overlaps between anterior and posterior cases, so does the anterior and central cases. Clearly, it is difficult to correctly classify these four sub-types. The augmented results based on each doctor’s classification are shown in Figure 6b–6d. There are still varying degrees of overlaps in the augmented datasets, such as the overlaps between central and posterior cases. However, more sub-clustering centers and” view point” images are yielded. The fewer overlaps between these data centers, the better the generated results will be.

### Network training results

First of all, we will show the results of training the network directly from the original 50 projected image. After that, the improvements caused by data augmentation will be presented subsequently. Finally, we compare the classification performance between deep learning based methods and our previous hand-crafted feature based method.

**Figure 7:**
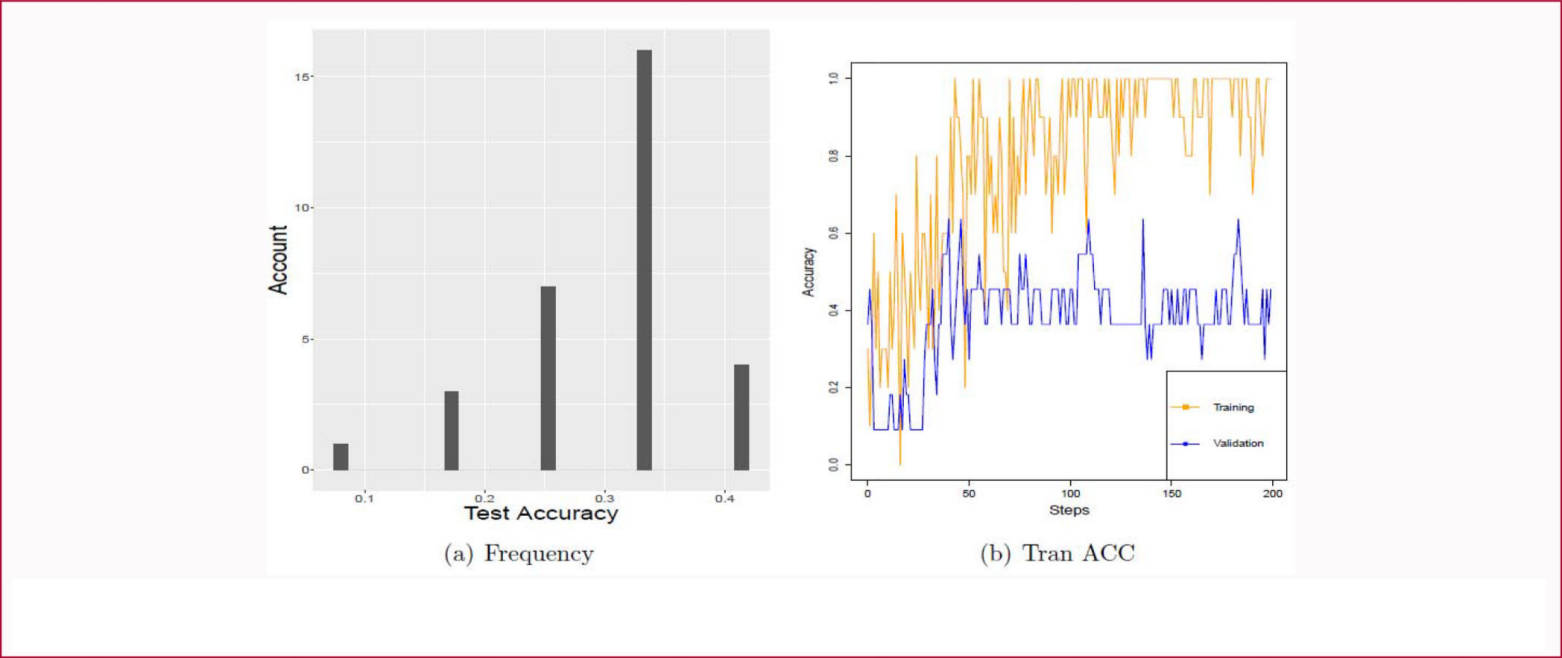
Testing, training and validation accuracy of Google Inception V3 on the original data. Panel a shows the testing accuracy of Google Inception V3 model fine-tuned by the raw data 30 times. Panel b shows the training (orange line) and validation accuracy (blue line).

We recorded the test accuracy of 30 experiments using the raw data directly to train the CNN models and found their test accuracy was less than 50% (Figure 7a). We draw one of the experiments’ training accuracy curve in Figure 7b. It showed that the CNN model was overfitted on these images because the training accuracy got up to 100% at the early steps and the validation accuracy fluctuated around 50%.

**Figure 8:**
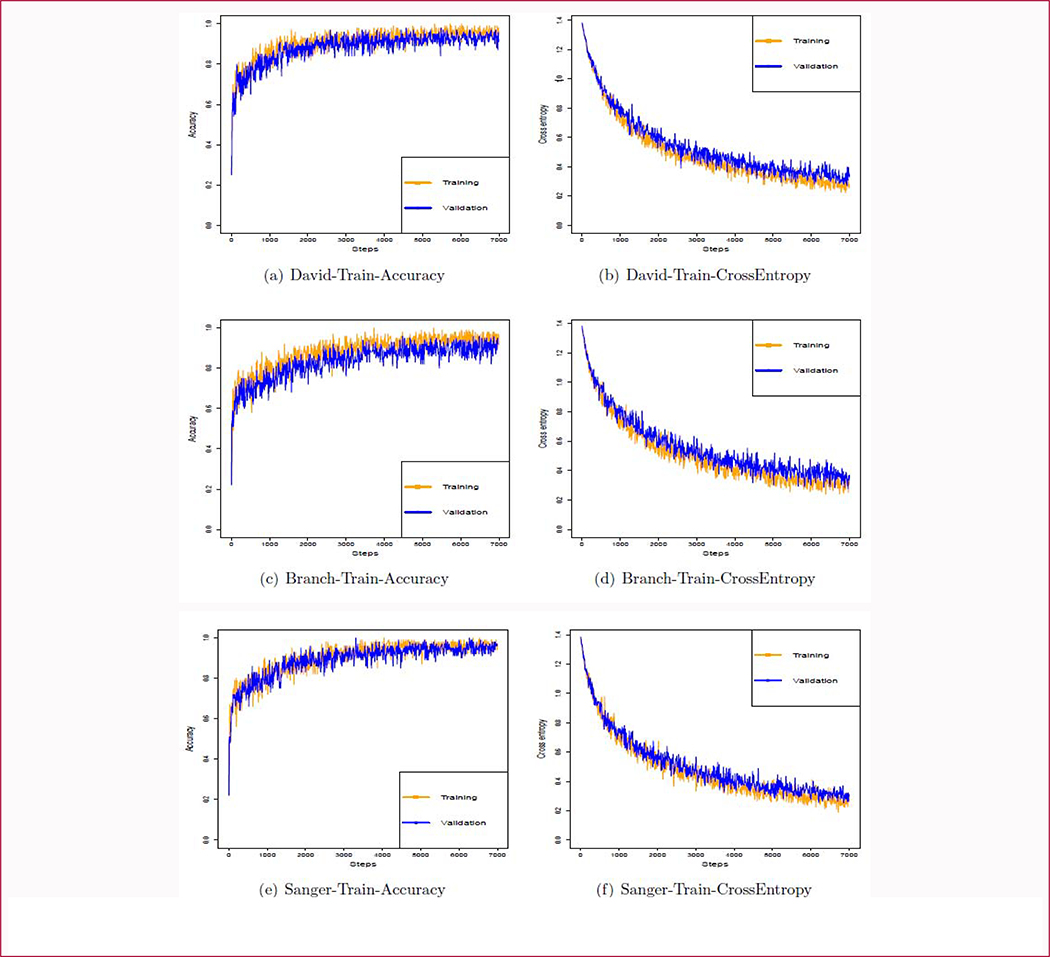
The transfer learning results based on different doctor’s labeling. The orange line indicates the training accuracy and the blue line indicates the validation accuracy.

The training and validation results after data augmentation are shown in Figure 8a,8c,8e. After 5000 iterations, the curves become flatter indicating that the CNNs have converged on their respective datasets. The training accuracy, validation accuracy and test accuracy of all these CNNs are over 94%, 90% and 90%, respectively (Table 1). Our results indicate that data augmentation can alleviate the problem of over-fitting caused by lack of data when fine-tuning a CNN model on a new field.

**Figure 9:**
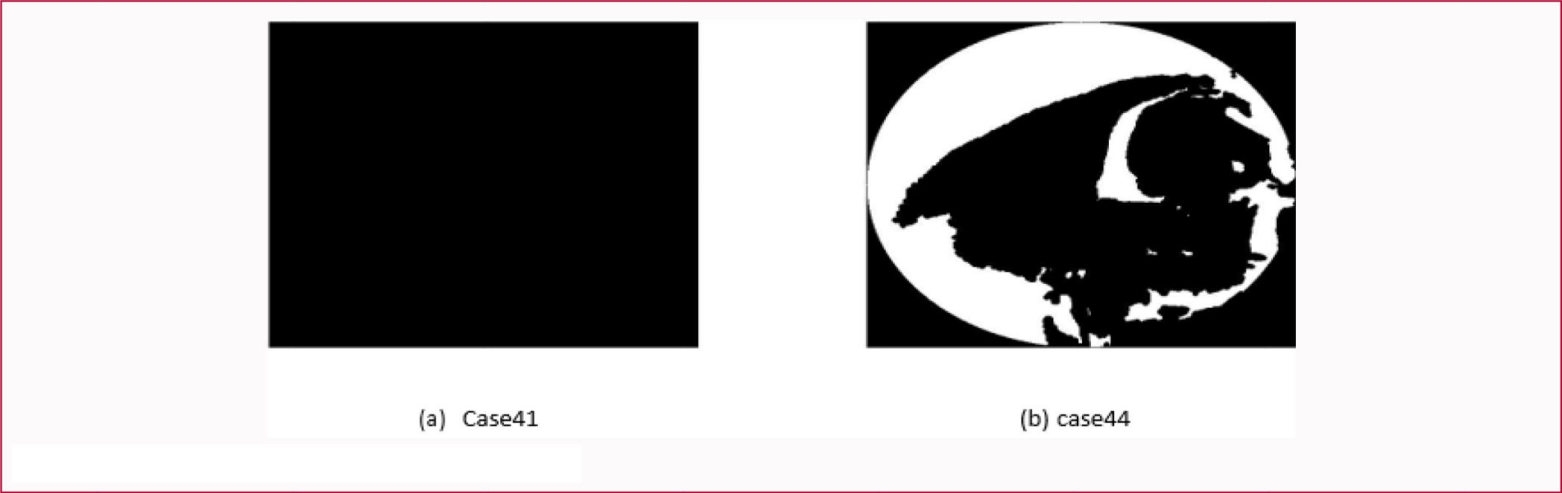
The wrongly classified cases by David-CNN.

Among these three CNNs, the Sarger-CNN outperformed the other two methods on the generated dataset. However, David-CNN had a much better performance than the other two CNNs in given the real unseen cases and achieved 75% accuracy when classifying 8 real unseen cases. Two wrongly classified cases by David-CNN are displayed in Figure 9. The 3D skull projection processing for those two cases was different from other cases because of irregular projecting images of these skulls. Our validation results indicate that different annotation on sagittal CSO cases can lead to different generalization performance.

Our deep learning-based model achieved more than 90% accuracy in model training, which is much higher than the training accuracy of our previous model based on the hand-crafted features-based method (72.7%). In term of the clinical classification of sagittal CSO, the deep learning model with physician’s annotation obtained a test accuracy of 75% on the 8 reserved real cases. Our results indicate that the deep learning-based method can learn more discriminative representations in the classification tasks of the sagittal CSO. In our study, if more cases were kept for the blind test, the number of cases for data augmentation and model training would be reduced, leading to the inability to fully utilize the learning capabilities of the deep learning model. In the future, if more and more researchers and physicians provide CSO data, deep learning-based models have potential to approximate or surpass the human’s performance in diagnosing CSO.

## Discussion

The main contribution of this study is that we applied the deep learning-based method in the task of sagittal CSO classification. By 3D skull image projecting, data augmentation, transfer learning and good annotations, the deep learning-based model outperformed the handcrafted feature-based model. The second contribution is that we proposed a data driven strategy for verifying the annotations among physicians. When the physicians couldn’t provide a united annotation on some disease with limit data, our data driven strategy could offer an objective reference in the view of data themselves. Finally, the model constructed based on the senior physician (David-CNN) obtained much better performance than these CNNs from two junior physicians on the real case images. It means that deep learning based classification system with senior physicians prior knowledge can be used to give the junior physicians new insights into the diagnosis of sagittal CSO.

Although several automated classification systems for CSO have been developed so far, only one study has focused on sub-classification of sagittal CSO using hand-crafted features- based model [29]. Considering the limitations of the hand-crafted features-based model in dealing high-dimension data, in this study, we developed three deep convolutional neural networks based on the manual classification results of CSO patients by different physicians and achieved an average prediction accuracy over 90% on the generated test data, which is higher than the accuracy from the hand-crafted features based model (72%). Comparing the hand-crafted feature-based model, our deep-learning-based models achieved high training and testing accuracy. However, when we applied our models to the real patient’s samples, the testing accuracy on the real unseen data varied greatly from 20% to 75%. The model constructed based on the senior physician (David-CNN) obtained much better performance than these CNNs from two junior physicians. Therefore, sample size and quality of annotation from physicians are key factors in establishing a practical mathematical model for clinical application.

From the physicians’ and our experimental classification results shown above, the classification of sagittal CSO subtypes still faces enormous challenges. We can see that there is no clear clustering center of subtypes and obvious overlaps between subtypes of sagittal CSO before data augmentation. Although more sub-clustering centers and “viewpoint” images were yielded after data augmentation, there is still lacking clear clustering center for each subtype of sagittal CSO. It may be due to i) classification variation from different doctors; and ii) limited available cases. Data shortages can lead to a biased distribution of machine learning model learning data. Different physicians use their own methods for diagnosing sagittal CSO, so it is difficult to obtain a unified annotation of sagittal CSO than a normal image classification task. This undoubtedly increases the difficulty of the training machine learning model. Another challenge comes from the image projection process. Two cases were misclassified showing in Figure 9 due to data pre-processing. As shown in Figure 9a, if the sutures of a skull are completely closed, the projected image will be black representing the area of the skull and no white sutures are displayed. The case showing in Figure 9b indicates that there is a large hole on the surface of the skull. In both cases, suture images can’t be well presented. Therefore, the current projecting method works better for regular sagittal CSO cases but not irregular ones. The projecting operation may have a large impact on features extraction or learning as the number of irregular cases increases.

Deep learning models have gained many impressive achievements in the field of image processing, natural language processing and machine translation in the past few years. Even without professional knowledge, many people can contribute to the annotation of general images. However, annotating medical images require strong expertise from the respective discipline. Labeling the medical dataset is a time-consuming process. It is difficult for physicians to annotate tens of thousands of medical images while focusing treatment of patients. Thus, the availability of labeled medical image datasets is limited. The 3D image projection may also lead to irregular 2D images that couldn’t be recognized by the CNN models. One possible solution is to learn 3D features directly from the 3D skulls of sagittal CSO patients with deep learning models or learn 2D features in the way of multi-view learning. By using the power of data and the data learning ability of deep learning models, these 2/3D features may fit the distributions of CSO data just like they did in the generated data in our experiments.

## Conclusion

In this study, we have applied a deep learning-based method to classify the four subtypes of sagittal craniosynostosis. 3D skull images, generated from computed tomography, were projected to a 2D plane. A data augmentation method was then used to increase the number of training images. Finally, a Google inception v3 model pre-trained on the ImageNet dataset was fine-tuned using the augmented images. The experiment results showed that our deep learning model based on data augmentation and transfer learning achieved an over 95% classification accuracy on the generated dataset, which outperformed our previous hand-crafted features-base method (72.7%). From the test results, we can see that the deep neural net- work not only succeed in learning a discriminative representation of the 3D skull projected images but also dealt with overfitting issue by data augmentation. Large amount of data is the main factor to train a deep learning model successfully on specific tasks. Although these three CNNs have a similar convergence rate, David-based CNN outperformed other models in the unseen data classification. It demonstrates that the doctor’s experience contributes a lot for successful diagnosis of sagittal CSO and for training a good deep learning model. Therefore, a well-trained model based on a large number of experienced physicians-labeled images can be a useful tool for doctors in the diagnosis of sagittal CSO.

Although the deep learning-based method achieved good classification results, the proposed algorithm is still affected by data pre-processing steps. For example, the 3D skull projection process may result in a black mask as complete closure of the cranial bones. At the same time, data shortage is still a critical problem when we want to learn 3D features from 3D skull directly. As a result, a proper data augmentation method is needed for 3D skull feature learning task. The GANs-based approach seems to be a good choice for 3D data augmentation issues. Finally, 3D CNN architectures cannot be directly applied in the sagittal CSO classification task since most of these models work on the CAD models in Object File Format (OFF). In the future, we will improve 3D-CNN or 3D-RNN based models to learn 3D high-level representations of the sagittal CSO for better classification.

## Figures and Tables

**Table 1:** The results of each CNN model on the Train,Validation and Test data.

Model	Training (generated)	Validation (generated)	Test (generated)	Test (real)
David CNN	96.25%	92.01%	**93.20%**	**75.00%**
Branch-CNN	94.86%	91.17%	92.50%	25.00%
Sarger-CNN	**97.16%**	**95.31%**	91.90%	37.50%
